# Artificial intelligence literacy and influencing factors among clinical nurses in southeastern China: a latent profile analysis

**DOI:** 10.1186/s12912-026-04872-w

**Published:** 2026-06-10

**Authors:** Jingyi Li, Yousheng Liu, Liting Xiang, Guanmian Liang

**Affiliations:** 1 Urology Department, Zhejiang Cancer Hospital, Hangzhou Institute of Medicine (HIM), Chinese Academy of Sciences, Hangzhou, Zhejiang 310022 China; 2 Nursing Department, Zhejiang Cancer Hospital, Hangzhou Institute of Medicine (HIM), Chinese Academy of Sciences, Hangzhou, Zhejiang 310022 China

**Keywords:** Latent profile analysis, Clinical nurses, Artificial intelligence literacy, Influencing factors

## Abstract

**Background:**

The rapid integration of artificial intelligence (AI) in clinical settings presents unprecedented opportunities, yet it concurrently triggers significant challenges, including ethical dilemmas, technology-induced anxiety, and care quality. Although clinical nurses are the primary users of healthcare AI, their literacy levels and cognitive profiles remain largely unexplored. This knowledge gap hinders the development of targeted interventions necessary to ensure the safe and effective deployment of AI in patient care.

**Objective:**

To identify the latent profiles of AI literacy among clinical nurses, explore the factors influencing profile membership, and provide insights for clinical nursing management.

**Methods:**

A cross-sectional study was conducted using a convenience sample of clinical nurses from 3 tertiary hospitals in southeastern China between December 2025 and February 2026. Data were collected using a sociodemographic questionnaire, the AI Literacy Scale, the Attitude Towards the Use of AI Technologies in Nursing Scale, and the Innovative Behavior Inventory. Latent profile analysis (LPA) was utilized to identify distinct AI literacy profiles, followed by univariate analysis and multivariate logistic regression to determine the factors associated with profile membership.

**Results:**

Of the 375 distributed questionnaires, 366 valid responses were obtained (response rate: 97.6%). The mean AI literacy score of the participants was 57.61 ± 11.27. LPA revealed three distinct latent profiles: the “low AI literacy-low use” group (21.00%), the “moderate AI literacy-high evaluation” group (71.60%), and the “high AI literacy-high ethics” group (7.40%). Multivariate logistic regression indicated that educational attainment, working experience, professional position, prior AI-related training, attitudes toward AI application, and innovative behavior scores were significant predictors of AI literacy profile membership (*P* < 0.05).

**Conclusion:**

While the overall AI literacy of clinical nurses in southeastern China is at a moderate-to-high level, significant heterogeneity exists within the population. Understanding these distinct profiles and their associated factors may inform the development of tailored educational strategies. Providing foundational support for low-literacy groups and encouraging advanced ethical discussions among highly literate groups could potentially assist nurses in adapting to the increasing integration of AI in clinical practice.

**Trial registration:**

This study does not involve clinical trials or interventional procedures and therefore does not meet the criteria for clinical trial registration.

## Introduction

Artificial intelligence (AI) refers to the application of computers and technology to replicate intelligent behavior and problem-solving akin to that of a human being [1]. AI has been applied across diverse sectors such as finance, commerce, entertainment, social media, and transportation [2]. The transformative impact of AI in nursing is manifested across a broad spectrum, including predictive analytics [3], decision-support systems [4], and robotic caregivers [5]. These technologies are fundamentally reshaping care delivery and patient engagement. Their integration into both practice and education is crucial for advancing health outcomes, personalizing care, and preparing nurses for an increasingly digital healthcare environment [6].

The accelerating adoption of AI in nursing practice necessitates a thorough investigation into the perceptions, competencies, and readiness of nurses to effectively interact with and leverage this transformative technology. AI literacy is the ability to understand, use, and critically evaluate AI applications. It involves recognizing AI, grasping how it works, using AI tools responsibly, and assessing their implications [7]. Higher levels of AI literacy present significant opportunities for success in technologically advanced settings. Conversely, limited skills in this area may pose substantial challenges to effective adaptation and engagement [8].

Current research indicates a notable gap between nurses’ level of AI literacy and their actual technological adoption behaviors. For instance, a descriptive study conducted in Turkey revealed that although nurses possessed a moderate level of AI literacy, their use of AI tools remained limited [9]. To date, research on AI literacy has primarily focused on the nursing education domain. Zhao et al. [10] demonstrated that nursing undergraduates in western China exhibited positive yet cautious attitudes toward AI, particularly regarding data privacy, tool reliability, and the impact on critical thinking skills. A study in Hunan Province similarly indicated that nursing students showed a moderate level of AI literacy, and that targeted AI education within a supportive environment can enhance their AI competency for clinical practice [11]. However, research specifically targeting clinical nurses’ AI literacy remains limited, leaving their precise competency levels and associated influencing factors inadequately understood. Therefore, it is necessary to elucidate the current state of AI literacy among clinical nurses and to identify the latent subgroups and related factors.

Previous research studies have shown that the use of technology predominantly depends on one’s knowledge of and attitude toward that technology, including AI [12]. A stronger educational background correlates with higher attitude scores, reflecting a more positive disposition toward AI. Furthermore, nurses with greater AI literacy tend to exhibit more favorable attitudes [13]. Concurrently, nursing professionals with an innovative mindset demonstrate greater efficacy in implementing AI technologies such as predictive analytics and Clinical Decision Support Systems (CDSS) [14].

Previous research on nurses’ AI adoption has primarily relied on traditional variable-centered approaches. While these studies clarify the relationships among isolated variables, they often mask critical individual variations and fail to capture how diverse characteristics combine within individuals. Given that AI adoption in nursing is influenced by a complex interplay of knowledge, attitude, education, and innovativeness, this study utilized Latent Profile Analysis (LPA) to identify heterogeneous profiles among nurses. LPA identifies latent subgroups within a sample based on patterns across multiple indicator variables, aiding in the detection of meaningful subgroups for targeted action [15]. Unlike variable-centered methods, LPA identifies distinct subgroups within a population based on response patterns across multiple indicators, providing an understanding of population heterogeneity and aiding in the development of profile-specific educational actions [16].

In this context, this study aimed to investigate the level of AI literacy among clinical nurses in southeastern China. It further sought to identify potential subgroups based on AI literacy characteristics and analyze the factors influencing these distinct profiles. The findings are intended to provide a theoretical basis for developing targeted training strategies, ultimately aiming to enhance nursing quality and advance the nursing discipline.

## Methods

### Design

A multicenter cross-sectional study was conducted following the STROBE guidelines.

### Participants

From December 2025 to February 2026, participants were selected from 3 hospitals in southeastern China via convenience sampling. The study included participants who satisfied the following criteria: (a) adult registered nurses (≥ 18 years); (b) clinical nursing experience of no less than 6 months. This threshold was not chosen arbitrarily. In Duchscher’s transition model, the first 3 months are characterized by an acute, task-focused “Transition Shock”; thereafter, novices enter the “Being” phase (months 4–9), a period of growing cognitive readiness and institutional familiarity [17]. The 6-month time point thus captures this theoretical shift and simultaneously coincides with the conclusion of the intensive 3- to 6-month orientation stipulated in China’s national standardized training guidelines [18]; (c) informed consent and voluntary participation in the study. Exclusion criteria included: (a) non-clinical positions; (b) nurses who were temporarily absent from their positions due to sick leave or maternity leave during the investigation period.

### Sample size

The sample size was estimated using the standard formula for a cross-sectional study with a continuous outcome variable: $\:\mathrm{N}={\left(\frac{{Z}_{1-{\upalpha\:}/2}\times\:\sigma\:}{{\updelta\:}}\right)}^{2}$. In this formula, $\:{Z}_{1-{\upalpha\:}/2}$ = 1.96, and $\:{\updelta\:}$ is the permissible margin of error, set at 1.28 for adequate precision. $\:\sigma\:\:$denotes the estimated population standard deviation of the AI literacy score (estimated at 11.27 based on our preliminary pilot study). To account for a potential 20% invalid response or attrition rate, the recruitment target was adjusted to approximately 373 participants. For the planned LPA, a minimum of 300 cases was sought to ensure robust profile detection. Ultimately, 375 questionnaires were distributed, yielding 366 valid responses, which satisfied the rigorous sample size demands for this study.

### Data collection

The survey was administered online via the “Questionnaire Star” platform (http://www.wjx.cn). Survey links were disseminated to nursing-specific WeChat groups in each participating hospital, along with a brief outline of the study purpose and completion instructions. Participants who provided informed consent could then independently access and submit the questionnaire via the provided link. The research was anonymous. We applied two criteria to screen out low-quality responses: uniform answers across all items and an online submission time shorter than 300 s.

### Measurement

#### Sociodemographic characteristics

Developed by the researchers through a comprehensive literature review, demographic data (gender, age, educational level), work-related information (department, professional title, professional position, monthly average number of night shifts, years of work experience) and AI related information (training status, AI usage frequency) were collected.

#### AI literacy scale

Nurses’ AI literacy was measured via the artificial intelligence literacy scale (AILS), which was developed by Wang et al. [19]. The scale comprises four subdimensions: Awareness, Use, Evaluation, and Ethics. The instrument utilizes a 7-point Likert scale, with total scores ranging from 12 to 84, positively correlating with the level of AI Literacy. The Cronbach’s α coefficient of AILS is 0.83 [19]. For this study, the Cronbach’s α coefficient was estimated to be 0.875.

#### Attitude scale towards the use of artificial intelligence technologies in nursing

The Attitude scale towards the use of artificial intelligence technologies in nursing (ASUAITIN) was developed by Dilek Yılmaz et al. [20] in 2024 to assess clinical nurses’ attitudes toward the use of AI in nursing practice. In this study, the Chinese version of the ASUAITIN translated by Hu et al. [21] was administered. The ASUAITIN consists of two subscales: a negative attitude subscale (6 items) and a positive attitude subscale (9 items). A higher total score (range: 15–75) corresponds to a more positive overall attitude regarding the application of AI in nursing practice [21]. The Cronbach’s α values for the two subdimensions are 0.890 and 0.932, respectively.

#### Innovative behavior inventory

Nurses’ Innovative Behavior was measured using the innovative behavior inventory (IBI), originally developed by Lukes and translated into Chinese in 2021 [22, 23]. The Chinese IBI includes 20 items across five dimensions: idea generation and search (6 items), planning and engagement (5 items), obtaining resources (3 items), overcoming obstacles (3 items) and clinical application (3 items). Each item is rated on a 5-point Likert scale, with higher scores (range: 20–100) indicating a greater level of innovative behavior [23]. The Cronbach’s α coefficient for the five subscales in this study varied between 0.857 and 0.920.

### Ethical considerations

This study was reviewed and approved by the Ethics Committee of Zhejiang Cancer Hospital [Ethics Approval No. IRB-2025-1718]. Given the use of an online survey platform, electronic informed consent was utilized. The first page of the online survey functioned as the consent form, detailing the study’s aims, participant rights, the voluntary nature of participation, and data confidentiality measures. Participants were required to check a mandatory box and click an “I agree to participate” button before they could access and complete the survey items. This mandatory electronic opt-in was recorded by the platform, serving as the electronic equivalent of documented written informed consent. All collected data were de-identified and maintained with strict confidentiality to safeguard privacy.

### Data analysis

The data were analyzed using Mplus 8.3 and IBM SPSS 29.0. A rigorous quality control protocol was implemented. Following initial collection, all questionnaires were reviewed independently by two researchers for completeness and logical integrity. Discrepancies or omissions identified were resolved by team consensus after verification against the source data. Following this procedure, the data were reviewed for completeness, cleaned to address any inconsistencies or missing values.

Potential profiles of resilience were distinguished via LPA. LPA is a model-based classification method designed to identify homogeneous subgroups within a heterogeneous sample [16]. This method is particularly valuable for capturing the subgroup differences in nurses’ AI literacy. LPA was conducted utilizing Mplus 8.3 software, with scores from the four dimensions of the AILS as exogenous variables. Starting from a one-class model, the number of classes in the model was gradually increased until the best­fitting model indicators were achieved. Model fit indices included the Akaike information criterion (AIC), Bayesian information criterion (BIC), and adjusted BIC (aBIC), as well as Entropy, Lo-Mendell-Rubin likelihood ratio (LMR), and Bootstrap likelihood ratio test (BLRT). Lower values of AIC, BIC, and aBIC indicated a better fit, while an Entropy value (range: 0–1) closer to 1 indicated more precise classification [24]. LMR and BLRT (*p* < 0.05) indicated that the k model was preferable to the k-1 model [25]. In addition, the interpretability of the profiles was also considered.

Participants were assigned to distinct latent classes, and subsequent statistical analyses were conducted using IBM SPSS 29.0. Descriptive statistics were used to analyze the sociodemographic characteristics and questionnaire data. Continuous variables were reported as means with standard deviations, and categorical variables were summarized as counts and percentages, as appropriate. Prior to the regression analysis, categorical variables were systematically coded. Ordinal variables were coded in an ascending numerical sequence: education level (1 = Technical secondary school, 2 = Junior college, 3 = Undergraduate, 4 = Masters or above), working age (1 = ≤ 5 years, 2 = 6–10 years, 3 = 11–20 years, 4 = > 20 years), and professional title (1 = Junior, 2 = Intermediate, 3 = Deputy senior). Nominal variables were coded with specific reference groups: “Position” used “None” as the reference category, creating indicator variables for the other specific roles; and “Whether receiving AI-related training” was binary-coded with “No” acting as the reference group (0 = No, 1 = Yes). Multiple logistic regression was employed to identify factors associated with latent class membership. The significance level was set at α = 0.05.

## Results

### Participant characteristics

A total of 375 clinical nurses participated in the study. 366 valid questionnaires were analyzed with an effective recovery rate of 97.6%. In this study, most of the participants were female (84.4%), and their average age was 33 years (SD = 5.59). The majority of the participants (73%) held a university degree, 66.9% of nurses had junior titles, and 44.3% had more than 10 years of experience. 19.1% nurses received AI-related training and 14.2% used AI daily in the past 6 months. See Table 1 for details.

**Table 1:** Sociodemographic results of nurses in different profile types (*N* = 366)

Variables	Total sample (*n* = 366)	C1(*n* = 77)	C2(*n* = 262)	C3(*n* = 27)	Statistical values	*P*
**Age**	32.79 ± 5.59	32.77 ± 4.90	32.79 ± 5.81	32.93 ± 5.51	0.009^c^	0.991
	n (%)	n (%)	n (%)	n (%)		
**Gender**					2.049^a^	0.359
Male	57(15.6)	8(10.4)	44(16.8)	5(18.5)		
Female	309(84.4)	69(89.6)	218(83.2)	22(81.5)		
**Education level**					38.835^b^	<0.001
Technical secondary school	25(6.8)	8(10.4)	17(6.5)	0(0.0)		
Junior college	74(20.2)	30(39.0)	42(16.0)	2(7.4)		
Undergraduate	243(66.4)	39(50.6)	186(71.0)	18(66.7)		
Masters or above	24(6.6)	0(0.0)	17(6.5)	7(25.9)		
**Working age (years)**					13.486^a^	0.036
≤ 5	78(21.3)	11(14.3)	58(22.1)	9(33.3)		
6 ~ 10	126(34.4)	37(48.1)	84(32.1)	5(18.5)		
11 ~ 20	125(34.2)	23(29.9)	90(34.4)	12(44.4)		
>20	37(10.1)	6(7.8)	30(11.5)	1(3.7)		
**Department**					4.525^a^	0.807
Internal Medicine	56(15.3)	13(16.9)	39(14.9)	4(14.8)		
Surgery	105(28.7)	18(23.4)	77(29.4)	10(37.0)		
Gynecology/Pediatrics	42(11.5)	10(13.0)	29(11.1)	3(11.1)		
ICU/Emergency	74(20.2)	15(19.5)	52(19.8)	7(25.9)		
Other	89(24.3)	21(27.3)	65(24.8)	3(11.1)		
**Night Shifts per Month**					0.399^a^	0.402
1 ~ 2	54(14.8)	8(10.4)	43(16.4)	3(11.1)		
3 ~ 5	161(44.0)	36(46.8)	110(42.0)	15(55.6)		
>6	96(26.2)	25(32.5)	66(25.2)	5(18.5)		
None	55(15.0)	8(10.4)	43(16.4)	4(14.8)		
**Professional Title**					9.704^b^	0.035
Junior	245(66.9)	56(72.7)	178(67.9)	11(40.7)		
Intermediate	108(29.5)	19(24.7)	74(28.2)	15(55.6)		
Deputy senior	13(3.6)	2(2.6)	10(3.8)	1(3.7)		
**Position**					42.966^a^	<0.001
(Deputy)Head Nurse/Nurse Manager Assistant/Secretary	201(54.9)	59(76.6)	138(52.7)	4(14.8)		
Nursing Team Leader/Clinical Instructor	48(13.1)	11(14.3)	34(13.0)	3(11.1)		
None	117(32.0)	7(9.1)	90(34.4)	20(74.1)		
**Whether receiving AI-Related Training**					10.062^a^	0.007
Yes	70(19.1)	10(13.0)	49(18.7)	11(40.7)		
No	296(80.9)	67(87.0)	213(81.3)	16(59.3)		
**Frequency of AI usage in the past 6 months**						
Daily	52(14.2)	13(16.9)	36(13.7)	3(11.1)	9.521^a^	0.300
At least once a week	92(25.1)	13(16.9)	70(26.7)	9(33.3)		
At least once a month	94(25.7)	23(29.9)	69(26.3)	2(7.4)		
Occasionally	68(18.6)	14(18.2)	47(17.9)	7(25.9)		
Almost never	60(16.4)	14(18.2)	40(15.3)	6(22.2)		
	M (P25, P75)	M (P25, P75)	M (P25, P75)	M (P25, P75)		
ASUAITIN	48.00(44.00,50.00)	45.00(42.00, 46.00)	48(45.00,50.00)	52(48.00,60.00)	58.454^d^	<0.001
IBI	77.00(60.00,83.00)	57.00(39.00, 64.00)	79(65.75,87.25)	80(77.00,97.00)	75.040^d^	<0.001

Note: ^a^chi-square tests; ^b^Fisher’s exact tests; ^c^analysis of variance (ANOVA); ^d^Kruskal‒Wallis H tests

### Results of latent profile analysis

LPA was conducted using the scores from the four dimensions of the AI Literacy Scale. Four models were tested, and their fit indices are shown in Table 2. As the number of latent profiles increased from 1 to 4, the values of AIC, BIC, and aBIC continuously decreased, although the magnitude of the reduction diminished at the 4-profile model. The AIC, BIC, and aBIC values were notably higher for the 2- profile model, yielding a classification too simplistic for clinical relevance. The entropy values for 2- and 3-profile were greater than 0.9, and 3-profile model presented the highest entropy value (0.953), indicating optimal accuracy. Conversely, the 4-profile model yielded a lower entropy (0.827) and a non-significant LMR (*P* = 0.067). Therefore, considering the proportion of each model and the practical significance of the results, we determined that the 3-profile model was the most reliable model.

**Fig. 1:**
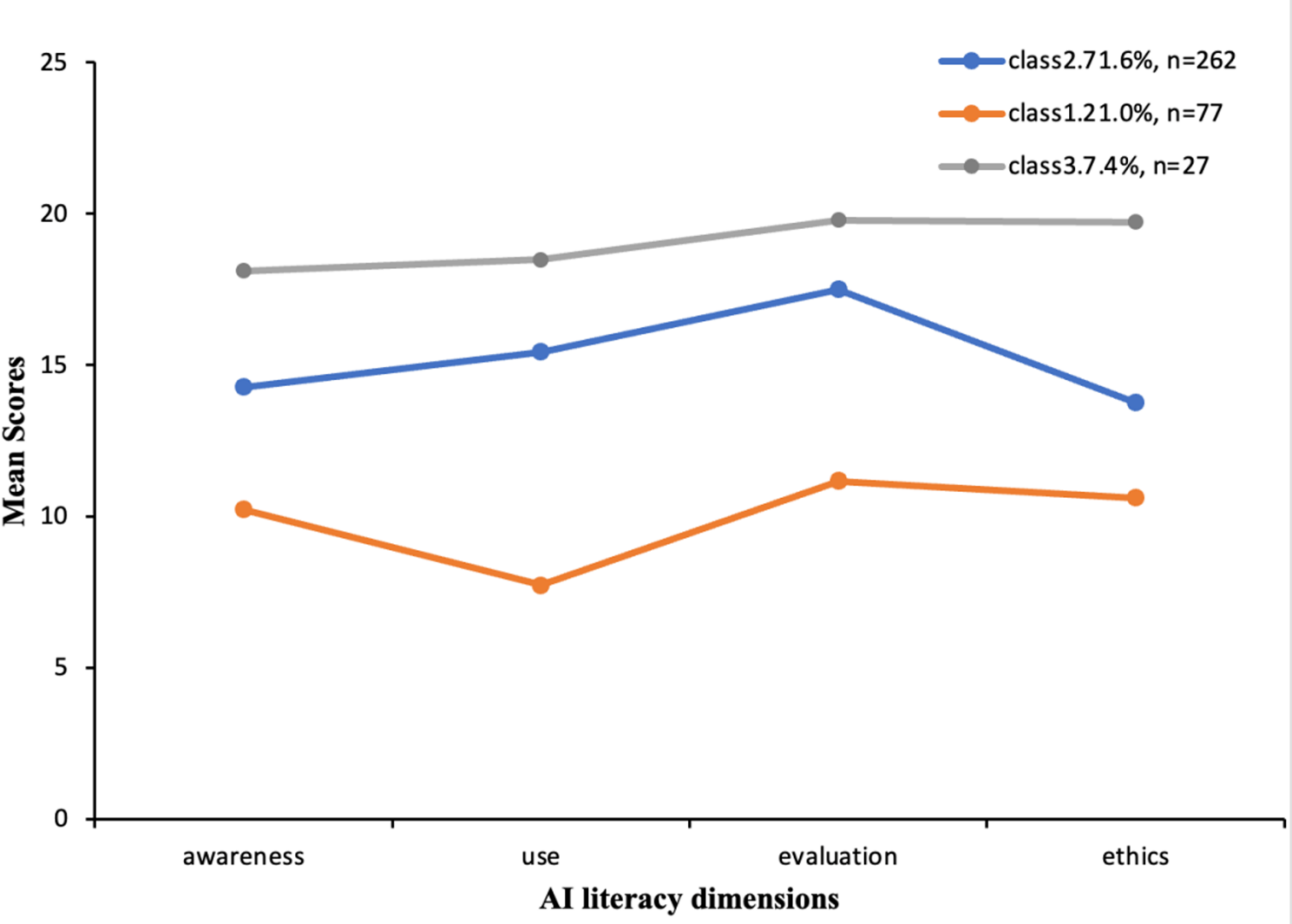
Latent profile analysis result of AI literacy(*n* = 366)

The characteristics of the three latent AI literacy profiles among clinical nurses are illustrated in Fig. 1. We labelled the profiles according to two criteria: overall AI literacy and the specific dimension showing a pronounced peak or divergent trajectory, which we regarded as the defining feature of that subgroup. According to the score of each dimension, the three categories were named as follows. Class 1 had the lowest scores on all dimensions of AI literacy, with a noticeable trough in the use dimension. Thus, it was named ‘Low AI literacy-low use group’, comprising 77 nurses (21.00%). Class 2 included 262 nurses (71.60%) and exhibited a moderate overall AI literacy level; however, Fig. 1 revealed a distinct peak in the evaluation dimension relative to their other competencies. Thus, it was named the ‘Moderate AI literacy-high evaluation group’. Class 3 comprised only 27 nurses (7.40%); this group achieved the highest scores across all dimensions. Notably, unlike the other two groups which demonstrated a downward score trend in the ethics dimension, Class 3 maintained a comparatively higher level of competency in this crucial area. Thus, it was named the ‘High AI literacy-high ethics group’.

**Table 2:** Information criteria fit statistics for one through four latent profiles

Model	AIC	BIC	aBIC	Entropy	LMR (*P*)	BLRT (*P*)	Probability (%)
1	7567.576	7598.797	7573.417	—	—	—	1
2	6880.290	6931.024	6889.780	0.951	<0.001	<0.001	0.781/0.219
3	6681.207	6751.454	6694.347	0.953	<0.001	<0.001	0.716/0.210/0.074
4	6603.878	6693.639	6620.669	0.827	0.067	0.072	0.302/0.454/0.182/0.061

Abbreviation: AIC = Akaike information criterion; BIC = Bayesian information criterion; aBIC = sample size-adjusted BIC; LMR-LRT = Lo‒Mendell‒Rubin likelihood ratio test; BLRT = bootstrap likelihood ratio test

### Factors influencing the latent profile membership

Univariate analysis revealed significant differences among the distinct latent profile groups in terms of educational level, working experience, professional title, position, history of AI training, attitudes toward AI application (ASUAITIN), and innovative behavior (IBI) scores (*P* < 0.05) (Table 1).

Prior to the multiple logistic regression analysis, a multicollinearity diagnosis was conducted to ensure the stability of the model. The diagnosis revealed that all independent variables exhibited variance inflation factor (VIF) values below 5 and tolerance values above 0.1, indicating the absence of significant multicollinearity among the predictors (Table 3). Multiple logistic regression analysis was performed using C1 (Low AI literacy-low use group) as the reference category (Table 4). The results indicated that nurses with a bachelor’s degree (*OR* = 1.757, 95%CI: 1.106–2.793), longer working experience (*OR* = 1.460, 95%CI: 1.018–2.093), and higher innovative behavior scores (*OR* = 1.054, 95%CI: 1.033–1.075) were significantly more likely to be classified into C2. Conversely, nurses holding administrative positions, such as head nurses or nursing managers (*OR* = 0.308, 95%CI: 0.121–0.780), had a significantly lower probability of belonging to C2 compared to frontline clinical nurses without administrative roles (*P* < 0.05). Furthermore, when comparing C3 to C1, nurses who had received AI-related training (*OR* = 22.279, 95%CI: 4.530-109.579), held a bachelor’s degree (*OR* = 16.432, 95%CI: 4.207–64.173), exhibited positive attitudes toward AI application (*OR* = 1.259, 95%CI: 1.087–1.457), and scored higher on innovative behavior (*OR* = 1.053, 95%CI: 1.006–1.102) were more likely to be categorized into C3. Similarly, nurses serving as head nurses or managers (*OR* = 0.021, 95%CI: 0.003–0.126) or nursing team leaders and clinical instructors (*OR* = 0.032, 95%CI: 0.004–0.269) were significantly less likely to be assigned to C3 compared to their non-titled frontline counterparts (*P* < 0.05). To delineate the transition from moderate proficiency to advanced mastery, a direct head-to-head comparison was conducted by designating C2 as the reference category. The analysis revealed that compared to C2, nurses holding a bachelor’s degree (*OR* = 9.350, 95% CI: 2.574–33.955), with prior AI-related training (*OR* = 11.502, 95% CI: 2.882–45.910), and exhibiting more positive attitudes toward AI application (*OR* = 1.248, 95% CI: 1.112–1.401) were more likely to elevate into the C3.

**Table 3:** Multicollinearity diagnosis of the independent variables

Variables	VIF	Tolerance
Education level	1.309	0.764
Working age (years)	1.336	0.748
Position	1.292	0.774
Professional Title	1.178	0.849
Whether receiving AI-Related Training	1.058	0.946
ASUAITIN	1.524	0.656
IBI	1.535	0.651

**Table 4:** Multiple logistic regression analysis of latent profiles of artificial intelligence literacy in clinical nurses

Variables	class 2: moderate AI literacy-high evaluation group (vs. class 1: low AI literacy-low use group)				class 3: high AI literacy-high ethics group (vs. class 1: low AI literacy-low use group)				class 3: high AI literacy-high ethics group (vs. class 2: moderate AI literacy-high evaluation group)			
	B	SE	OR (95%CI)	*P*	B	SE	OR (95%CI)	*P*	B	SE	OR (95%CI)	*P*
**Position (ref: None)**												
Head Nurse/Manager Assistant/Secretary	-1.179	0.475	0.308(0.121–0.780)	0.013	-3.886	0.926	0.021(0.003–0.126)	< 0.001	-2.707	0.808	0.067(0.014–0.325)	< 0.001
Team Leader/Clinical Instructor	-1.363	0.598	0.256(0.079–0.826)	0.023	-3.440	1.086	0.032(0.004–0.269)	0.002	-2.077	0.929	0.125(0.020–0.775)	0.025
**Whether receiving AI-Related Training (ref: No)**												
Yes	0.661	0.427	1.937(0.839–4.471)	0.121	3.104	0.813	22.279(4.530-109.579)	< 0.001	2.443	0.706	11.502(2.882–45.910)	< 0.001
Education level	0.564	0.236	1.757(1.106–2.793)	0.017	2.799	0.695	16.432(4.207–64.173)	< 0.001	2.235	0.658	9.350(2.574–33.955)	< 0.001
Working age (years)	0.378	0.184	1.460(1.018–2.093)	0.040	0.423	0.461	1.526(0.619–3.765)	0.359	0.044	0.431	1.045(0.449–2.432)	0.918
Professional Title	-0.160	0.304	0.852(0.470–1.547)	0.599	0.995	0.657	2.706(0.746–9.809)	0.130	1.155	0.603	3.174(0.974–10.349)	0.055
ASUAITIN	0.008	0.049	1.008(0.916–1.110)	0.867	0.230	0.075	1.259(1.087–1.457)	0.002	0.222	0.059	1.248(1.112–1.401)	< 0.001
IBI	0.052	0.010	1.054(1.033–1.075)	< 0.001	0.051	0.023	1.053(1.006–1.102)	0.028	-0.001	0.022	0.999(0.957–1.042)	0.958

Abbreviation: SE = Standard Error; OR = Odds Ratio; 95% CI = 95% Confidence Interval

## Discussion

### Current status and heterogeneity of AI literacy

Our findings indicate that the overall AI literacy of clinical nurses in southeastern China is at a moderate-to-high level. The mean AI literacy score of the participants was 57.61 ± 11.27. This aligns with recent studies targeting nursing populations [9, 26], suggesting a steady improvement in nurses’ understanding of AI technologies driven by the advancement of healthcare. From the perspective of the domestic and international environmental context, this proficiency reflects China’s accelerated national push towards “Smart Hospital” infrastructure, which has substantially increased frontline nurses’ exposure to AI tools, such as predictive analytics and intelligent monitoring systems. However, the situation differs significantly in other countries. In the United States, the American Association of Colleges of Nursing (AACN) has integrated health informatics into its core competencies [7]. Europe, on the other hand, focuses on comprehensive digital literacy frameworks. Compared with these regions, specialized AI courses in China’s nursing education are still in a transitional stage [27].

Furthermore, the LPA results reveal significant internal heterogeneity within this population, successfully identifying three distinct latent profiles. Notably, the “Moderate AI literacy-high evaluation group” constitutes the majority (71.60%). This suggests that while Chinese nurses recognize the value of AI within their highly digitized hospital environments, they often lack the structured support seen in international frameworks to master these applications fully. In contrast, the “High AI literacy-high ethics group” accounts for a mere 7.40%. This small proportion suggests that comprehensive nursing talents equipped with a solid understanding of AI ethical issues may still be limited in number. While international discourse emphasizes stringent data privacy increasingly, standardized ethical guidelines specific to nursing AI applications are still nascent domestically. Existing hospital training programs in China prioritize the operational efficiency of new technologies while neglecting complex interdisciplinary ethical considerations [12].

### Influencing factors of latent profiles

From a socio-technical perspective, the integration of AI in healthcare is not merely a technical implementation but a complex reorganization of social roles and clinical workflows. Our study identified higher educational attainment, prior AI training, positive attitudes, and innovative behaviors as protective factors that enhance nurses’ AI literacy. These findings are highly consistent with the Technology Acceptance Model (TAM) and recent international literature [28, 29], which emphasize that cultivating an innovative mindset and providing structured training are vital for personnel to adapt to technological shifts.

Recent international discourse on digital health advocates for “digital leadership” and robust informatics competencies among nursing managers to guide technology adoption and reduce risks [30]. It is generally assumed that nursing administrators, possessing higher comprehensive competencies and better access to resources, should demonstrate superior information literacy. Nevertheless, this study yielded a significant finding that contradicts some existing literature: nurses holding administrative positions or clinical instructor roles exhibited lower levels of AI literacy compared to frontline clinical nurses without administrative duties.

Today’s smart hospitals are becoming highly digital. Frontline nurses are the direct users of clinical smart devices, and this high-frequency usage compels them to improve their AI operational skills rapidly. Conversely, nursing administrators devote much of their time to managerial duties, such as staff scheduling, quality control, and resource allocation. Consequently, they experience less frequent exposure to novel clinical AI tools. Furthermore, their demanding administrative workloads leave them with limited time and energy to participate in systematic AI education. Finally, generational differences may play a role. Although age did not enter the final regression model, administrators typically have more seniority, and when confronted with AI technologies, they may exhibit more technological conservatism compared to younger frontline nurses [31]. International studies emphasize that successful digital transformation in healthcare requires managers who are not only administratively proficient but also digitally fluent [32]. If nursing leaders lack corresponding AI knowledge, they may struggle to safely guide the rapid adoption of AI tools by frontline staff. Addressing this gap may support the more responsible and effective use of AI in patient care.

### Implications for future clinical practice

Based on the profile characteristics and influencing factors identified, nursing administrators may consider developing targeted educational or support strategies. For C1 (Low literacy group), foundational AI literacy campaigns are essential to mitigate technology anxiety, emphasizing the practical benefits in alleviating daily nursing workloads. For C2 (Moderate literacy group), the training should focus on proper application while deepening education in AI ethics, data security, and decision-making. For nursing administrators, hospitals could consider incorporating AI ethics and leadership elements into advanced training programs. While administrators do not need to become AI experts, they should develop the strategic vision to evaluate the clinical value of AI tools from a top-level perspective and lead the digital transformation of the nursing team.

### Limitations

The cross-sectional design means that despite identifying associations via logistic regression, we cannot draw definitive causal conclusions regarding AI literacy. Longitudinal tracking is therefore needed to confirm these dynamics. Additionally, relying on a convenience sample from tertiary hospitals in China’s southeastern region restricts the broader applicability of our results. Since healthcare informatics are highly advanced in this specific area, these patterns might differ significantly in primary care facilities or less developed regions.

Another constraint is the reliance on self-reported data. Nurses’ self-assessments of their innovative behaviors and AI attitudes are susceptible to social desirability bias, which could artificially increase their scores. Therefore, subsequent qualitative investigations would be helpful to investigate the authentic psychological experiences and barriers during the application of AI technologies.

We also recognize that our 6-month clinical experience inclusion threshold may not fully account for the entire professional socialization process of novice nurses. Under the Chinese Standardized Training Outline for Newly Recruited Nurses, this whole transition takes up to two years. Thus, some participants were still in the early stages of standardized training. Their limited depth of practical clinical experience could influence their evaluations of complex, high-stakes AI decision-support tools, a factor that should be acknowledged as a potential limitation.

The same caution applies to the ‘High AI literacy-high ethics group’, which accounted for only 7.4%. Because this segment is so small, any conclusions specific to it remain tentative, and we would urge caution in extending its predictors or traits to the wider nursing workforce.

## Conclusions

Based on our latent profile analysis, clinical nurses in southeastern China generally demonstrate moderate to high AI literacy. However, significant heterogeneity exists within the population, which can be categorized into three distinct latent profiles: the “Low AI literacy-low use group,” the “Moderate AI literacy-high evaluation group,” and the “High AI literacy-high ethics group.” We also found that nurses with higher educational backgrounds, previous AI training, positive attitudes toward technology, and strong innovative tendencies were more likely to belong to the higher AI literacy profiles. Therefore, recognizing these distinct profiles and their associated factors may provide a descriptive reference for designing continuing nursing education. Tailored educational strategies that consider the characteristics of each profile could support the nursing workforce in adapting to modern clinical environments.

## Data Availability

The data supporting the conclusions of this research will be made available from the corresponding authors upon reasonable request.

## Abbreviations

AI: Artificial intelligence
LPA: Latent profile analysis
CDSS: Clinical decision support systems
AILS: Artificial Intelligence Literacy Scale
ASUAITIN: Artificial intelligence technologies in nursing
IBI: Innovative behavior inventory
AIC: Akaike information criterion
BIC: Bayesian information criterion
aBIC: Adjusted BIC
LMR: Lo-Mendell-Rubin likelihood ratio
BLRT: Bootstrap likelihood ratio test
TAM: Technology Acceptance Model
